# Iodine nutritional status is not a direct factor in the prevalence of the *BRAFV600E* mutation in papillary thyroid cancer

**DOI:** 10.20945/2359-3997000000530

**Published:** 2023-01-17

**Authors:** Yan-Yu Lin, Yu-Shan Hsieh

**Affiliations:** 1 Taipei Medical University Hospital Division of Endocrinology and Metabolism Department of Internal Medicine Taipei City Republic of China (Taiwan) Division of Endocrinology and Metabolism, Department of Internal Medicine, Taipei Medical University Hospital, Taipei City, Republic of China (Taiwan); 2 National Taipei University of Nursing and Health Sciences School of Nursing Taipei City Republic of China (Taiwan) School of Nursing, National Taipei University of Nursing and Health Sciences, Taipei City, Republic of China (Taiwan); 3 Taipei Medical University Hospital Department of Research Taipei City Republic of China (Taiwan) Department of Research, Taipei Medical University Hospital, Taipei City 11031, Republic of China (Taiwan)

**Keywords:** Papillary thyroid carcinoma, *BRAFV600E*, Iodine

## Abstract

**Objectives:**

Papillary thyroid carcinoma (PTC) accounts for approximately 85%-90% of all thyroid cancers. Of the iodine-metabolizing genes, *BRAFV600E* is a highly specific target for PTC and may have a reciprocal causative relationship with iodide-metabolizing genes.

**Materials and methods:**

In this study, we performed a data analysis of selected quantitative studies to determine the relationship between iodine nutritional status and the prevalence of the *BRAF600E* mutation in patients with PTC. Five studies were selected for meta-analysis based on the selection criteria.

**Results:**

A total of 2,068 patients were divided into three groups: low (urinary iodine concentration [UIC] < 100 μg/L), adequate (UIC 100-200 μg/L), and high (UIC ≥ 200 μg/L). The results were obtained using RevMan software, and the pooled odds ratios (ORs) were calculated using Mantel-Haenszel statistics with a 95% confidence interval (CI). The OR for the prevalence of the *BRAFV600E* mutation between the high and adequate groups was 1.25 (95% CI 0.64-2.43, *p* = 0.51), and the OR between the low and adequate groups was 0.98 (95% CI 0.42-2.31, *p* = 0.96). The *BRAFV600E* mutation risk did not change significantly at different levels of iodine nutrition ( *p* = 0.33) in statistical analyses.

**Conclusion:**

We conducted preliminary research on dietary iodine intake and the *BRAFV600E* mutation in PTC. The results suggested that abnormal iodine intake might not directly influence the prevalence of the *BRAFV600E* mutation in these patients. Further research into the associations between dietary iodine intake and the *BRAFV600E* mutation in PTC, including the underlying mechanisms, is required.

## INTRODUCTION

Studies on the possible influence of iodine nutritional status on iodine-metabolizing gene mutation in patients with papillary thyroid carcinoma (PTC) have been inconclusive. PTC accounts for approximately 85%-90% of all thyroid cancers ( 1 ). Among the iodine-metabolizing genes, the *b-raf* oncogene ( *BRAF* ) mutation caused by thymine-to-adenine transversion is the major oncogenic genetic alteration and a highly specific target for PTC, and the valine-to-glutamic acid mutation ( *BRAFV600E* ) is the most prevalent. One Korean study reported that patients with the *BRAFV600E* mutation who received treatment exhibited a higher frequency of the more aggressive pathological features of PTC ( 2 ). Iodine is involved in the occurrence of the *BRAFV600E* mutation in normal and tumour tissues, which might provide crucial information on tumorigenesis. Relatively low and extremely high iodine intake are associated with an increased probability of thyroid cancer ( 3 ). Because the *BRAFV600E* mutation and iodide-metabolizing genes may have a reciprocal causative relationship, the association between changes in iodine intake and expressions of related downstream genes should be considered.

Taiwan was an iodine-deficient area in the past, and a mandatory salt iodization policy was implemented from 1971 to 2002. A 1997-2002 study in Taiwan ( 4 ) revealed a high prevalence (73%) of the *BRAFV600E* gene mutation in conventional PTCs, which was similar to the levels reported in South Korea (73.7%), an iodine-replete area ( 5 ). An epidemiological study in South Korea reported that a relatively low (<300 μg/L) and extremely high urinary iodine concentration (UIC; ≥500 μg/L) were both significant risk markers for *BRAFV600E* mutations in the thyroid ( 2 ). Iodine nutritional status could affect the prevalence of various thyroid diseases, such as hyperthyroidism. However, the relationship between iodine nutritional status and *BRAFV600E* in patients with PTC remains controversial and unclear.

Various studies have been carried out on the relationship between the prevalence of *BRAF600E* and PTC, but few have considered the relationship between iodine nutritional status and the prevalence of *BRAF600E* in patients with PTC. In the present study, we performed a literature review and data analysis on selected quantitative studies to clarify the role of iodine nutritional status in the prevalence of the *BRAF600E* mutation in patients with PTC.

## MATERIALS AND METHODS

**Figure 1:**
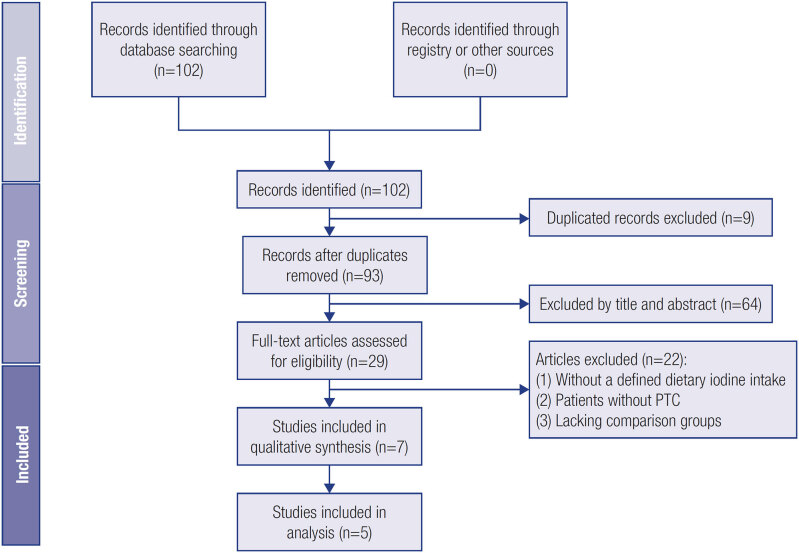
Flowchart of the selection of eligible studies.

Figure 1 shows a flowchart detailing the selection of eligible studies.

### Eligibility criteria

Inclusion and exclusion criteria were defined at the time of the study conception and before data collection. Studies were included if the following criteria were met:

1. Design: comparative studies that were randomized controlled trials (RCTs) or non-RCTs published as full-length articles in English in peer-reviewed journals between 2009 and 2020.
2. Participants: patients with PTC.
3. Interventions and exposure: abnormal iodine nutritional status was the main exposure.
4. Comparison: adequate iodine nutritional status.
5. Outcome: prevalence of the *BRAF* mutation.

We excluded studies without ( 1 ) a defined dietary iodine intake, ( 2 ) patients with PTC, and ( 3 ) comparison groups. Studies included in the analysis were reviewed for the following characteristics: author and year of publication, language of publication, and definition of iodine intake.

### Information sources and search strategy

Two independent researchers searched the MEDLINE, PubMed, and EMBASE databases for English language articles from inception to 2020 using the following terms: “iodine”, “ *BRAFV600E* ”, and “PTC” or “papillary thyroid cancer.” A total of 102 potential articles published between 2009 and 2020 were identified, and their abstracts were reviewed. Although no restriction was made in terms of geographical region and study duration, because of time restrictions, studies published in the English language and indexed up to 2020 were included.

### Study selection

Articles were initially screened for relevance using their titles and abstracts. After removing duplicate and irrelevant articles, a full text review was performed on the retrieved articles based on the selection criteria. One author (YSH) performed the initial screening and selection of all the papers, including the quality assessments. Two authors (YSH and YYL) conducted the quality assessments independently, which were then rechecked by the first author (YYL). The quality assessments were based on the following characteristics: representativeness of the participants (selection bias), study design, data collection methods, and completeness of the outcome data. Figure 1 summarizes the screening process and number of studies excluded and retrieved. A total of 102 articles were retrieved from the databases. We selected 29 articles based on their abstracts and performed a holistic review of the text; seven studies were selected according to eligible criteria. However, one article was excluded because it lacked information on urine iodine levels, and another article lacked a comparison group. Finally, we included five studies, which comprised 2,086 participants.

### Definition of iodine intake

Median UIC is the most commonly used indicator of population iodine nutrition. UIC, as a population-level indicator of iodine status, has also been recommended for this purpose by the World Health Organization (WHO). According to the WHO, levels of dietary iodine intake based on UIC are as follows: low dietary iodine (UIC < 100 μg/L), adequate dietary iodine (UIC 100-200 μg/L), and high dietary iodine (UIC ≥ 200 μg/L).

### Data synthesis

We compared the prevalence of the *BRAFV600E* mutation between groups with various levels of iodine intake. The results were obtained using Review Manager Version 5.3.5 (RevMan for Windows, 2015; The Cochrane Collaboration, Oxford, UK), and the pooled odds ratios (ORs) were calculated using Mantel–Haenszel statistics with a 95% confidence interval (CI). The I^2^ test was used to quantify heterogeneity. If the I^2^ test indicated high heterogeneity (I^2^ > 50%) between studies, a random-effect model was selected; *p* < 0.05 was considered statistically significant. To quantify and summarize our data, we used the OR for the prevalence of the *BRAFV600E* mutation in a particular group to analyse our results.

### Statistical analysis

For a further analysis of our results, we used a between-group analysis to explore the relationship between the groups. This method allowed us to identify changes in the *BRAFV600E* mutation in relation to the groups’ iodine nutritional status. The results are presented as means ± standard deviation. The results for *BRAFV600E* mutation prevalence (%) were analysed using a one-way analysis of variance (ANOVA), and other variables were compared using ANOVAs followed by a least significant difference post hoc test using SPSS Statistics version 22 (IBM, Armonk, NY, USA). A *p* value of <0.05 was considered statistically significant.

## RESULTS

A total of 2,086 PTC patients with various levels of dietary iodine intake (UIC < 100 μg/L group = 553, UIC 100–200 μg/L group = 772, UIC ≥ 200 μg/L group = 761) were included. Five studies were based on PTC patients with different levels of iodine dietary intake, and two studies were excluded because they lacked iodine intake data or comparison groups ( 6 ). Table 1 summarizes the characteristics of the included studies. After excluding unqualified articles, the remaining seven were included in the final review, with five quantitative studies included in the meta-analyses.

**Table 1:** Summary of included studies

Authors (Country, year)	Study type	Patients (n)	Age (mean)	Criteria of UIC (μg/L)	BRAFV600E mutation (%)
Guan (China, 2009) ( 7 )	Non-RCT	HI:559	43.59	≥900 a	387/559 (69)
		AD1:240		188 a	128/240 (53)
		AD2:76		198 a	38/76 (50)
		LI:157		82.77 a	86/157 (55)
Vuong (Japan, Vietnam, 2016) ( 8 )	Non-RCT	HI:67	49.3	≥281 b	55/67 (82)
		LI:53	43.5	56 b	44/53 (83)
Kim (Korea,2018) ( 2 )	Non-RCT	VHI:75	46	≤500	64/75 (85)
		HI:27	48	300-499	15/27 (56)
		AD:33	46	200-299	28/33 (85)
		RL:49	45	100-199	40/49 (82)
		LI:31	44	≤100	26/31 (84)
Pellegriti (Italy, 2009) ( 9 )	Non-RCT	AD:106	N	ISA (113.8 ± 8.4) c	68/205 (33)
		LI:205		IDA (18.9 ± 4.1-43.2 ± 4.9) c	55/106 (52)
Liu (China, 2014) ( 10 )	Non-RCT	HI:202	N	High (198) d	148/202 (73)
		AD:206		Normal (82.77) d	102/206 (50)
Lee (Korea, 2018) ( 11 ) e	Single group study	HI:210	43.4-45.1	Mutation (+): 884	169/210 (89)
				Mutation (-):792.9	

RCT: randomized controlled trial; VHI: very high iodine intake group; HI: high iodine intake group; AD: adequate iodine intake group; RL: relatively low iodine intake group; LI: low iodine intake group; N: not obtained.

a The level of UIC base on the mean UIC from the population in the area. AD1 group is the population from Shenyang, China (Median UIC is 188 μg/L), AD2 group is the population from Shanghai, China (Median UIC is 198 μg/L) and LI group is the population from Qingdao, China (Median UIC is 82.77 μg/L).

b The level of UIC in Vietnamese and Japanese based on previously study.

c ISA and IDA(iodine sufficient area and iodine-deficient area, the level of UIC is based on previous study).

d High and normal intake area, the level of UIC based on previously study.

e Reference 10 is excluded the reference in present study because of the single group study which have no compared population.

### Prevalence of the *BRAFV600E* mutation with UIC levels

**Figure 2:**
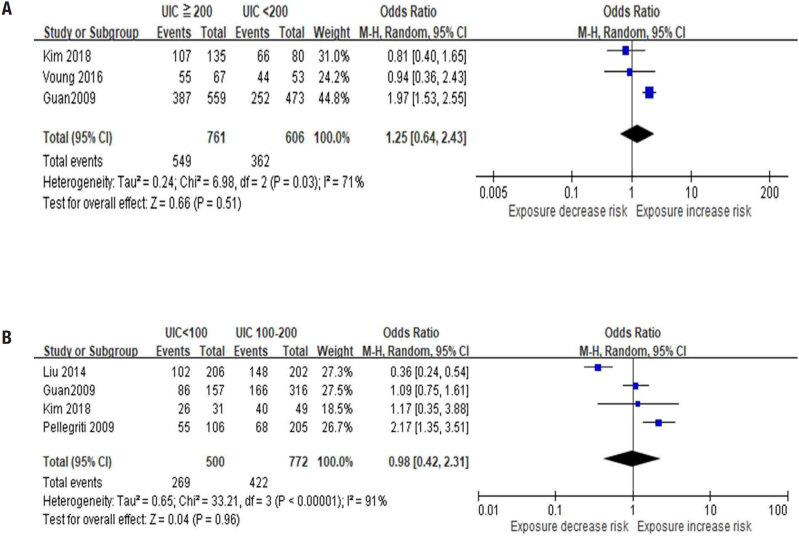
( **A** ) Forest plot of the between-group analysis of different levels of iodine nutritional status (UIC < 200 μg/L and ≥ 200 μg/L) in relation to the prevalence of the *BRAFV600E* mutation. ( **B** ) Forest plot of the between-group analysis of different levels of iodine nutritional status (UIC < 100 μg/L and 100-200 μg/L) in relation to the prevalence of the *BRAFV600E* mutation. UIC: urinary iodine concentration; CI: confidence interval; M-H: Mantel-Haenszel; *BRAF* mutation: *BRAFV600E* mutation.

First, we analysed the prevalence rate of the *BRAFV600E* mutation between the high (UIC ≥ 200 μg/L) and adequate (UIC < 200 μg/L) status groups. The OR between these groups was 1.25 (95% CI 0.64-2.43, Figure 2A ). In the UIC ≥ 200 µg/L group, the OR for the *BRAFV600E* mutation was not significantly different from that of the UIC < 200 µg/L group ( *p* = 0.51).

Based on these results, we compared the prevalence rates of the *BRAFV600E* mutation in the low- (UIC < 100 μg/L) and adequate- (UIC ≥ 100 μg/L and ≤ 200 μg/L) status groups. The OR between these groups was 0.98 (95% CI 0.42-2.31, Figure 2B ), and no significant difference ( *p* = 0.96) was identified. The estimated heterogeneity I^2^ variance in the data was 71% ( Figure 2A ) and 91% ( Figure 2B ). The estimated heterogeneity variance between the studies was 92%, mainly resulting from the study by Kim and cols.( 2 ). However, the I^2^ test revealed a considerable level of heterogeneity in the risk estimates.

### The prevalence of the *BRAFV600E* mutation at UIC levels

Because the results demonstrated relatively high heterogeneity between articles, we further analysed the prevalence of the *BRAFV600E* mutation in patients with high, adequate, and low iodine intake separately.

**Figure 3:**
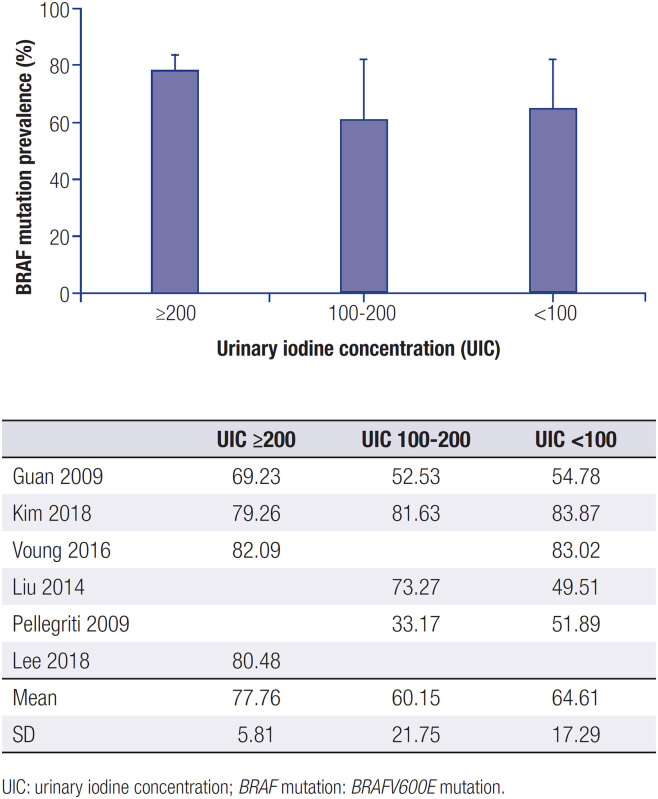
Statistical analyses of the *BRAFV600E* mutation according to different levels of UIC.

The five articles that included iodine nutritional status were initially included. However, the *BRAFV600E* gene mutation risk between the three groups was not significantly different ( *p* = 0.3288; Figure 3 ).

## DISCUSSION

Does iodine nutritional status influence the iodine-metabolizing gene mutation involved in PTC? In fact, the influence of abnormal iodine nutritional intake on the *BRAFV600E* mutation in patients with PTC remains controversial.

Hence, we tried to clarify the role of iodine nutritional status in this pathway. Compared with other studies on healthy populations, we focused on the *BRAFV600E* mutation in patients with PTC because, compared with healthy populations, these patients may have more consistent characteristics related to PTC. To our knowledge, this is the first literature study on the association between iodine nutritional status and *BRAFV600E* mutation in PTC, and it could help correctly evaluate the effect of iodine on *BRAFV600E* mutation. However, similar ethnic backgrounds and biological characterizations must be considered to clarify the association further.

Neither relatively high nor low iodine intake demonstrated a significant influence in the prevalence of the *BRAFV600E* mutation between groups. Although some studies have indicated that abnormal iodine nutritional status may play a role in the tumorigenesis of PTC and influence the increase in *BRAFV600E* mutations in patients with PTC ( 2 , 7 , 9 ), our results suggested that dietary iodine intake might not directly influence the *BRAFV600E* mutation.

A large cohort study in China investigated the association between iodine intake and *BRAF* mutation in various cities. The results revealed that lymph node metastasis and disease progression were significantly associated with *BRAFV600E* mutation and high iodine intake ( 7 ), but another report revealed no statistical difference in *BRAFV600E* -mutation prevalence between Japanese and Vietnamese residents ( 8 ). Additionally, in a study from Canada, the mutation rate of the *Ras* oncogene (a *BRAF* upstream gene mutation) was significantly higher in an iodine-deficient area, with rates of 85% versus 17% in adenomas and 50% versus 10% in follicular carcinomas. However, a similar result was not identified in relation to PTC ( 12 ). This suggests iodine intake may induce *BRAFV600E* mutation through various pathways in patients with PTC. Differences in geographical areas, diet preference, and culture may be major variables affecting the analysis results. In the present study, the I^2^ heterogeneity test also revealed a considerable level of heterogeneity.

**Figure 4:**
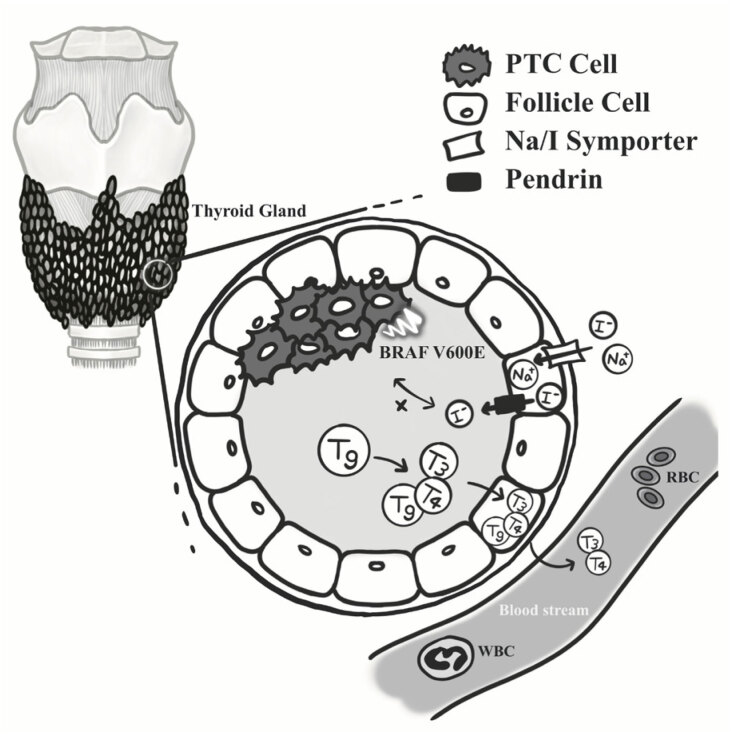
Schematic representation of iodide metabolism. Iodine nutritional intake may modulate the level of thyroid hormone by NIS, pendrin or Tg. However, iodine intake may induce the *BRAFV600E* gene mutation through different, indirect, or undiscovered pathways in patients with PTC.

TSH: thyroid stimulating hormone; *BRAF* : the *b-raf* oncogene; ERK: the extracellular-signalregulated kinase, also known as the Ras-Raf-MEK-ERK pathway; Tg: thyroglobulin; T3: triiodothyronine; T4: thyroxine; NIS: sodium-iodine symporter; TPO: thyroperoxidase; PTC: papillary thyroid carcinoma.

*BRAFV600E* mutation in patients with PTC was reported to be associated with the abnormal expression of key genes involved in iodine metabolism ( 6 ). A schematic of iodine metabolism in the thyroid is presented ( Figure 4 ). In the membrane of thyroid cells, the sodium-iodine symporter (NIS), an integral membrane glycoprotein, mediates iodine transportation into the thyroid follicular cells to induce thyroid hormone biosynthesis, subsequently decreasing in the thyroid follicular cells to produce T3 and T4 ( 13 ). The iodine resulting from the degradation of T3 and T4 re-enters circulation and is eventually excreted in urine; in addition, iodide could also induce the downregulation of NIS in thyroid cells ( 14 ).

The pathway between the *BRAFV600E* mutation and PTC is complex, and NIS might play a key role. A Brazilian study identified NIS gene suppression in conventional PTC, which is related to *BRAFV600E* mutation ( 15 ). This might indicate that the *BRAFV600E* mutation was modulated by NIS in PTC. The molecular pathways of *BRAFV600E* mutation in patients with PTC are numerous and complex; one study reported that these pathways were able to influence NIS function ( 16 ). Moreover, in vitro results have indicated that during *BRAFV600E* expression, suppressed iodine-metabolizing genes were identified in thyroid cell lines ( 17 ). Another study recruited a cohort of Taiwanese patients with PTC and compared a group with the *BRAFV600E* mutation with a wild-type group, demonstrating that *BRAFV600E* -mutated PTC involved 27 exclusive pathways ( 18 ). Therefore, further research to clarify the role and underlying mechanism of NIS in *BRAFV600E* -mutated PTC may be necessary.

This study has some limitations. First, in the past 10 years, few studies have investigated this subject, and some of the included studies might not have adequately adjusted for potential confounding risk factors. High heterogeneity may be present when estimated using the I^2^ statistic if the number of studies is small, which is considered the main reason for the high heterogeneity in the present study. Second, in some of the included studies, the determination of UIC was based on previous results or published statistical data, not on participant data. Third, the dietary iodine levels varies from region to region, and some researchers have not established whether their work was conducted on a high- or low-level iodine population. Thus, we separated the research groups and used a between-group analysis method and statistical analysis to identify evidence as the basis for further research. Further research is required, but the limitations of this study should be considered. Although some previous studies have linked iodine intake and the *BRAF* mutation, little empirical evidence exists for a direct relationship between the two factors in relation to patients with PTC.

In conclusion, we conducted preliminary research on dietary iodine intake and the *BRAFV600E* mutation in patients with PTC. The results suggested that abnormal iodine intake might not directly influence the prevalence of the *BRAFV600E* mutation in these patients. However, iodine intake may induce *BRAFV600E* gene mutation through various indirect pathways in patients with PTC. Further studies on potential mechanisms underlying the associations between dietary iodine intake and *BRAF* mutation in patients with PTC will provide additional insights into PTC.
